# Predicting Survival of *De Novo* Metastatic Breast Cancer in Asian Women: Systematic Review and Validation Study

**DOI:** 10.1371/journal.pone.0093755

**Published:** 2014-04-02

**Authors:** Hui Miao, Mikael Hartman, Nirmala Bhoo-Pathy, Soo-Chin Lee, Nur Aishah Taib, Ern-Yu Tan, Patrick Chan, Karel G. M. Moons, Hoong-Seam Wong, Jeremy Goh, Siti Mastura Rahim, Cheng-Har Yip, Helena M. Verkooijen

**Affiliations:** 1 Saw Swee Hock School of Public Health, National University of Singapore and National University Health System, Singapore, Singapore; 2 Department of Surgery, Yong Loo Lin School of Medicine, National University of Singapore and National University Health System, Singapore, Singapore; 3 Department of Medical Epidemiology and Biostatistics, Karolinska Institutet, Stockholm, Sweden; 4 National Clinical Research Centre, Kuala Lumpur Hospital, Kuala Lumpur, Malaysia; 5 Julius Center for Health Sciences and Primary Care, University Medical Center, Utrecht, the Netherlands; 6 Department of Hematology Oncology, National University Cancer Institute, National University Health System, Singapore, Singapore; 7 Department of Surgery, Faculty of Medicine, University of Malaya, Kuala Lumpur, Malaysia; 8 Department of Surgery, Tan Tock Seng Hospital, Singapore, Singapore; 9 Yong Loo Lin School of Medicine, National University of Singapore and National University Health System, Singapore, Singapore; 10 Ministry of Health Holdings, Singapore, Singapore; 11 Department of Radiology, University Medical Center Utrecht, Utrecht, the Netherlands; Health Canada and University of Ottawa, Canada

## Abstract

**Background:**

In Asia, up to 25% of breast cancer patients present with distant metastases at diagnosis. Given the heterogeneous survival probabilities of *de novo* metastatic breast cancer, individual outcome prediction is challenging. The aim of the study is to identify existing prognostic models for patients with *de novo* metastatic breast cancer and validate them in Asia.

**Materials and Methods:**

We performed a systematic review to identify prediction models for metastatic breast cancer. Models were validated in 642 women with *de novo* metastatic breast cancer registered between 2000 and 2010 in the Singapore Malaysia Hospital Based Breast Cancer Registry. Survival curves for low, intermediate and high-risk groups according to each prognostic score were compared by log-rank test and discrimination of the models was assessed by concordance statistic (C-statistic).

**Results:**

We identified 16 prediction models, seven of which were for patients with brain metastases only. Performance status, estrogen receptor status, metastatic site(s) and disease-free interval were the most common predictors. We were able to validate nine prediction models. The capacity of the models to discriminate between poor and good survivors varied from poor to fair with C-statistics ranging from 0.50 (95% CI, 0.48–0.53) to 0.63 (95% CI, 0.60–0.66).

**Conclusion:**

The discriminatory performance of existing prediction models for *de novo* metastatic breast cancer in Asia is modest. Development of an Asian-specific prediction model is needed to improve prognostication and guide decision making.

## Introduction

Global incidence rates of breast cancer are on the rise and the increase is largely due to an upsurge in breast cancer rates in Asia [1]. Asian women are more likely to be diagnosed with late stage disease compared to their Western counterparts. Approximately 10% to 25% of Asian breast cancer patients present with *de novo* metastatic disease, compared to 3% to 5% in Europe and United States [2], [3], [4], [5], [6]. In addition, metastatic lesions in Asian women are larger and often involve multiple sites [7].

Metastatic breast cancer is incurable. Median survival rates range from one to four years, but on an individual level, survival times of up to 15 years have been reported [8], [9], [10], [11], [12], [13], [14], [15]. While recent studies suggest that surgical removal of primary breast tumor has a positive impact on the survival of *de novo* metastatic patients [16], [17], [18], systemic therapy, is the main treatment. Due to advances in loco-regional and systemic treatment and due to the detection of small, solitary metastases, survival has improved over time, especially in patients with hormone receptor-positive tumors [12], [15].

Accurate assessment of individual prognosis of patients with *de novo* metastatic breast cancer is needed for treatment decision making. In addition, like all patients with cancer, women with distant metastases want to know their prognosis [19]. As clinicians are known to be overoptimistic in predicting survival [20], prediction rules can be useful for this heterogeneous group of patients with different treatment options. Although many multivariable prognostic indices have been developed for breast cancer in the last two decades, the majority are not applicable to patients with *de novo* metastatic disease [21], [22], [23]. In this study, we aim to identify prediction tools which can be used for prognostication of patients with *de novo* metastatic breast cancer and externally validate their performance in the Singapore-Malaysia hospital-based breast cancer registry.

## Materials and Methods

### Ethics statement

This study obtained ethics approval from National Healthcare Group (NHG) Domain Specific Review Board (DSRB).

### Systematic review

Our first step was to perform a systematic review of the available literature, according to the PRISMA guidelines [24]. A free text search was performed on 13 August 2013 to identify eligible studies using MEDLINE and EMBASE electronic database. Our search strategy included search terms and synonyms for prognostic models and the following string was used: ((metastatic breast cancer) AND ((prognostic scor* OR prognostic index OR nomogram OR predictive model OR validation OR validate OR prognostic model OR predictor) AND (scor* OR index OR model OR predict* OR nomogram OR validat*))) NOT (expression profiling OR microarray* OR proteomic OR affymetrix). After reviewing the titles and abstracts, full text was selected applying predefined in- and exclusion criteria. Included were studies presenting multivariable models, with the aim to predict overall survival of metastatic breast cancer patients. We excluded animal models or clinical trials on treatment efficacy, as well as studies which used disease free, progression free survival or response to treatment as the only outcome of interest. Etiological studies which only assessed the effect size of one specific prognostic factor or only evaluated the prognostic value of a single biomarker were not included. We also excluded prediction tools developed for patients with metastases from various primary cancers. Prognostic tools for patients with advanced cancer nearing the end of life or tools specific for recurrent metastatic breast cancer were not included as these patients have been exposed to multiple chemotherapy regimens and are often treatment resistant. Two studies which validated previously published models in metastatic breast cancer patients were excluded. Additional articles were retrieved by cross-referencing. Details regarding the author, year of publication, study design, model variables and performance measures were extracted if available. Quality of the selected publications was assessed using items listed in the Strengthening the Reporting of Observational Studies in Epidemiology (STROBE) statement, which were relevant to our study [25].

### Validation set

Validation of the performance of the selected prediction models was performed within the Singapore Malaysia Hospital Based Breast Cancer Registry. This registry consists of three hospital-based breast cancer registries in Singapore and Malaysia. National University Hospital (NUH) and Tan Tock Seng Hospital (TTSH) are two public tertiary hospitals in Singapore. The registry at NUH includes cases diagnosed between 1990 and 2010 while the TTSH registry started in 2001. University Malaya Medical Centre (UMMC), an academic tertiary hospital in Kuala Lumpur, Malaysia, has prospectively collected breast cancer cases from 1993 to 2008. All three registries include data on basic patient demography, clinical and pathological tumor characteristics and treatment profile. These registries have received approval from respective ethical review committees. Death information was obtained from the hospitals' medical records and ascertained by linkage to National Registration Departments in both countries. Patients were followed up from the date of diagnosis until the date of death or date of last contact whichever came first. The date of last contact was 1 November 2010 for UMMC patients, 1 July 2011 for NUH patients and 1 October 2012 for TTSH patients. Details of the registries have been described previously [3], [4], [26]. Breast cancer patients with distant metastasis detected within three months after diagnosis were identified from this registry and formed the basis of this study. Individual data on the date of birth, ethnicity, tumor size, grade, estrogen receptor (ER) status, progesterone receptor (PR) status, human epidermal growth factor receptor 2 (HER2) status, site(s) of metastasis and treatment were available in the registry. For NUH patients we went back to the clinical files as site(s) of metastasis was not systematically recorded. Due to the lack of information on hormone receptor status in the early years, we restricted our cohort to women diagnosed between 2000 and 2010. Patients with metastases in the ipsilateral supraclavicular lymph nodes but no metastasis at any other distant site were not considered as metastatic patients, according to the sixth edition of the tumor node metastasis classification of the American Joint Committee on Cancer (AJCC) [27].

### Statistical analysis

In the validation set, we investigated the pattern of missing data and assumed that data missingness was related to at least one other variable but not dependent on value of the observation itself, i.e. missing at random [28]. A total number of 230 (36%) individuals had complete data on all variables used in validation and 90 (14%) cases had 3 or more variables missing. On average, each individual had 1.13 variables missing (standard deviation = 1.22), ranging from 0 to 5. Missing values were imputed once using regression imputation [28].

For each individual patient, we calculated the prognostic score for the different prognostic models/indices except for those developed by recursive partitioning analysis [29] and artificial neural network [30], as terminal nodes were missing in our dataset or algorithm was not provided to allow calculation of prognostic scores. For models including performance status, a variable that was not captured in our database, we assumed all patients to be fit at the time of diagnosis, i.e. 0 on Zubrod scale, which is the same as the Eastern Cooperative Oncology Group (ECOG) and the WHO scale, and 100 on the Karnofsky performance status (KPS) scale. In order to check this assumption, we retrieved comorbidity data from the medical records of a subset of 87 NUH patients who diagnosed after 2006. We also assumed the best case scenario for lactate dehydrogenase (LDH). For brain metastasis models, a score of zero (best case scenario) was assigned to the largest brain metastasis dimension in Marko et al.'s model. We assumed no trastuzumab use for HER2 positive patients in Ahn et al.'s model, as in Singapore and Malaysia trastuzumab use was rare during the time of our study. Since our study population consisted of patients who were metastatic at presentation, disease free interval (DFI) was set as zero for all women.

The distribution of each prognostic score was then divided into tertiles with the exception for Rabinovich's model, for which were only two possible combinations. We compared the survival of low, intermediate and high-risk score patients by plotting the Kaplan Meier survival curves for each tertile. Median survival and 95% confidence intervals (CI) were obtained for different groups and differences were tested by log-rank test and log-rank test for trend. The discrimination ability of the models was assessed by concordance statistic (C-statistic), which is the probability of correctly distinguishing between deceased and surviving patients within a random pair of patients [31]. The interpretation of C-statistic is equivalent to area under a curve (AUC) in receiver operating characteristic (ROC) analysis. A value of 0.5 indicates no discrimination and value of 1.0 means perfect discrimination. For models with C-statistic larger than 0.6, 1-year, 2-year and 3-year cumulative survival probabilities were plotted for each quintile of the prognostic score to test calibration.

## Results

### Systematic review

**Figure 1:**
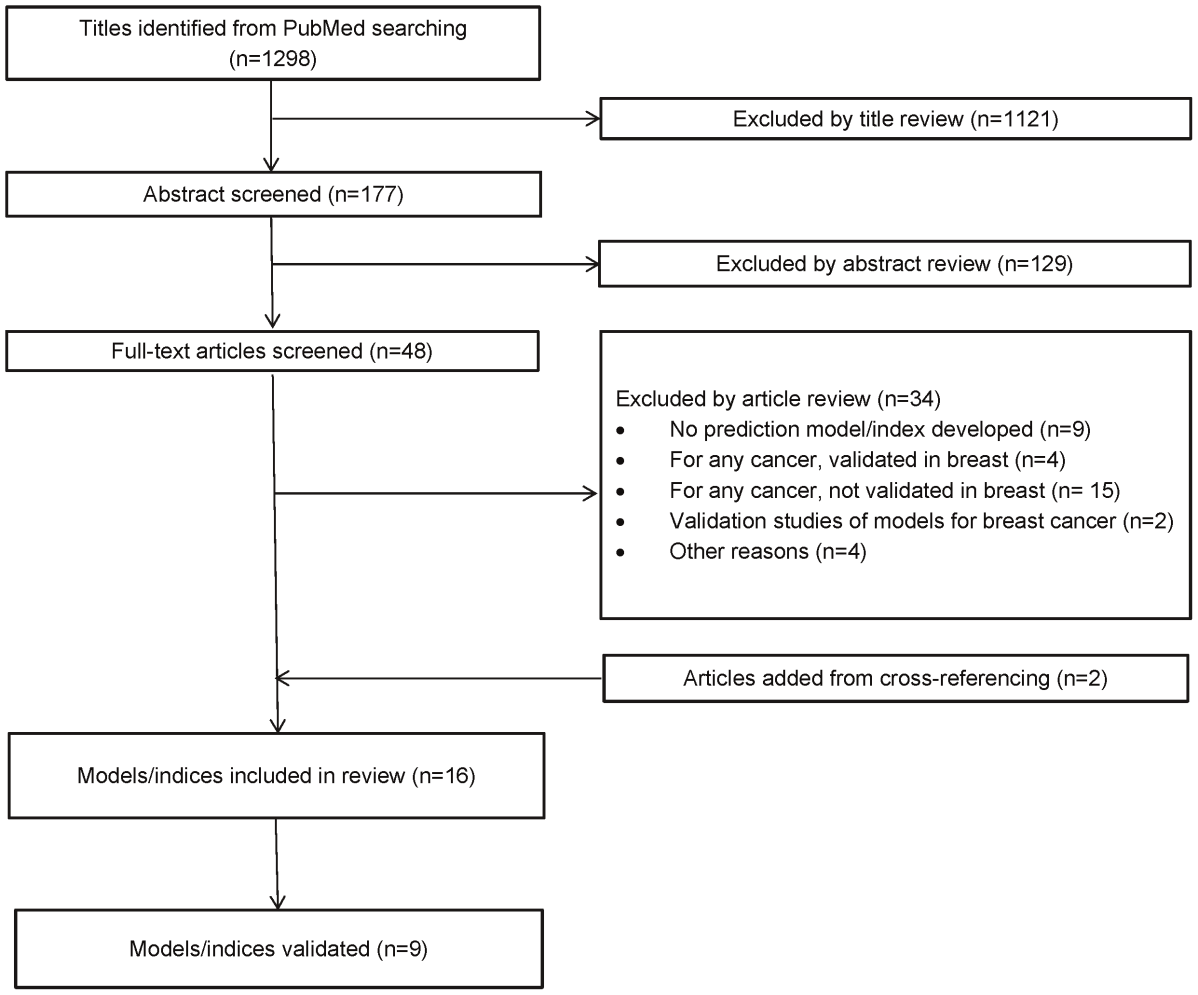
Flow chart of study selection process. n =  number of studies.

The search strategy resulted in 1298 titles (Figure 1). Forty-eight full text articles were selected after screening the titles and abstracts and two articles were added by cross-referencing. A total of 16 prognostic indices met our inclusion criteria. Eight models were developed for patients with metastatic breast cancer in general, seven for patients with brain metastasis from breast cancer and one for breast cancer patients with metastatic spinal cord compression [32], [33], [34], [35], [36], [37], [38], [39], [40], [41], [42], [43], [44], [45], [46], [47]. All prognostic indices were designed for both *de novo* and recurrent metastatic breast cancer patients (Table 1). Study sizes ranged from 83 to 619 patients, with a median study size of 246 patients. The median survival from time of detection of metastasis ranged from 9.6 to 22 months. Cox regression incorporated time-to-event data and all-cause mortality as outcome was used for model development in 13 studies. Three studies conducted recursive partitioning analysis and one used artificial neural network. For Cox regression modeling, forward or backward stepwise selection with different cut-off P-values, either 0.05 or 0.1 was applied to identify final predictors.

**Table 1:** Study characteristics of prognostic models for metastatic breast cancer patients.

Authors	Year of publication	Number of patients	Country	Setting	Period of diagnosis	Median survival	Predictors	Analysis	Discrimination	Validation
Nash et al.	1980	138	USA	Single institution	1973–1977	17 months	age, number of metastatic site(s)	Cox Regression	Not reported	No
Hortobagyi et al.	1983	619	USA	Single institution	1973–1976	22 months	LDH, PS,site(s) of metastasis, radiotherapy, ALKP and extent of disease	Cox Regression	Not reported	Temporal
Williams et al.	1986	191	UK	Single institution, patients without brain metastasis	1974–1984	Not reported	Grade, ER status, DFI, site(s) of initial metastasis	Cox Regression	Not reported	External and temporal
Rabinovich et al.	1992	362	Argentina	Multiple institutions	1978–1985	21 months	PS, visceral involvement	Cox Regression	C-statistic = 0.72	Temporal
Yamamoto et al.	1998	233	Japan	Multiple institutions	Not available	21.5 months	adjuvant chemotherapy, presence of distant lymph nodes, liver metastasis, LDH and DFI	Cox Regression	Not reported	External
Ryberg et al.	2001	469	Denmark	single institution	1983–1992	14.7 months	Metastatic site(s), LDH, age, ER status and PS	Cox regression	Not reported	Temporal
Giordano et al.	2011	311	USA	Single institution	2004–2009	34.0, 28.3, 20.5 and 8.1 months for four risk groups based on CTC	ER, PR, HER2 status, visceral metastasis, bone metastasis, number of metastatic site(s), therapy type, line of treatment; and CTC count	artificial neural network	C-statistic = 0.73	Internal
Giordano et al.	2013	236	USA	Single institution	2002–2009	Not reported	age, hormone receptor and HER2 status, visceral metastases, PS and CTC	Cox Regression	C-statistic = 0.74	External
Le Scodan et al.	2007	117	France	Single institution, patients with brain metastasis	1998–2003	5 months	RTOG RPA, Lymphocyte count, hormone receptor status	Cox Regression	Not reported	No
Nieder et al.	2009	83	Norway, Germany	2 institutions, patients with brain metastasis	2002–2007	16.0, 5.5 and 2.7 months for low, medium and high risk groups	KPS, extracranial metastases, multiple brain metastasis and DFI	Cox Regression	Not reported	No
Sperduto et al.	2012	400	USA	11 institutions, patients with brain metastasis	1993–2010	13.8 months	KPS, age, ER, PR and HER2 status	Cox regression, RPA	Not reported	External
Ahn et al.	2012	171	Korea	Single institution, patients with brain metastasis	2000–2008	9.6 months	KPS, extracranial metastases, age, trastuzumab, ER, PR and HER2 status	Cox Regression	Area under a curve = 0.73	Internal and external
Marko et al.	2012	261	USA	Single institution, patients with brain metastasis	1999–2008	16.2 months	age, KPS, Non-CNS and number of CNS metastases, largest dimension brain metastasis, ER, PR, HER2, breast cancer stage	Cox Regression	C-statistic = 0.67	Internal
Le Scodan et al.	2012	130	France	Single institution, patients with brain metastasis	1998–2006	7.43 months	KPS, age, trastuzumab, ER,PR,HER status and lymphocyte count	RPA	Not reported	No
Niwińska et al.	2012	441	Poland	Single institution, patients with brain metastasis	2003–2009	7 months	KPS, number of brain metastases and extracranial metastasis	RPA	Not reported	No
Rades et al.	2013	255	Germany, Netherland, UK, Bosnia Herzegovina	Multiple institutions, patients with metastatic spinal cord compression	1995–2011	Not reported	PS, ambulatory status, other bone metastases, visceral metastases, interval to radiotherapy, time of developing motor deficits	Cox Regression	Not reported	Internal

Abbreviation: LDH, Lactate dehydrogenase; PS, Performance status (Zubrod/ECOG/WHO score); ALKP, alkaline phosphatase; DFI, disease free interval; KPS, Karnofsky performance score; CNS, Central nervous system; ER, Estrogen receptor; PR, Progesterone receptor; HER2, Human epidermalgrowth factor receptor 2; CTC, circulating tumor cells; RTOG, Radiation Therapy Oncology Group; RPA, recursive partitioning analysis.

Performance status, ER status, metastatic site(s) and disease free interval were the most common prognostic factors included in the different models. Performance status was measured on different scales, i.e. five studies used Zubrod/ECOG/WHO score while 6 models for brain metastasis used KPS [33], [35], [37], [39], [41], [42], [43], [44], [45], [46], [47]. Model coefficients or hazard ratios were presented in all Cox regression models. Six studies transformed the model into a scoring system for easy calculation of predicted survival and three studies developed a nomogram [32], [36], [37], [39], [41], [42], [43], [44], [47]. Recursive decision tree was constructed from recursive partitioning analysis in two studies [48], [49]. Only 5 studies evaluated the discrimination of their models using C-statistic or AUC [35], [38], [39], [43], [44], which ranged from 0.67 to 0.74 (moderate discrimination). Calibration was assessed by plotting predicted versus observed survival for only two models, which turned out to be well calibrated [43], [44]. Four studies conducted internal validation using random subset of data, ten-fold cross-validation and bootstrapping with 200 and 1000 resamples [38], [43], [44], [47], [49]. Temporal validation of the model using data collected from the same hospital but later than those in the development set was conducted in four studies [33], [35], [37]. Five models were externally validated in other hospitals or outside the original country [36], [39], [43], [44], [48]. Quality of the selected publications is summarized in Table 2.

**Table 2:** Summary of quality assessment of publications selected for validation.

Authors	Inclusion and exclusion criteria clearly described	Outcome (survival) clearly described	Predictors clearly described	Loss of follow-up <20%	Characteristics of patients clearly described	Discrimination & calibration	Internal or external validation
**Nash et al.**	Y			Y	Y		
**Hortobagyi et al.**		Y	Y	Y	Y		Y
**Williams et al.**	Y	Y	Y				Y
**Rabinovich et al.**	Y	Y	Y	Y	Y	Y	Y
**Yamamoto et al.**		Y	Y	Y	Y		Y
**Ryberg et al.**			Y		Y		Y
**Giordano et al. 2011**	Y		Y		Y	Y	Y
**Giordano et al. 2013**	Y	Y			Y	Y	Y
**Le Scodan et al. 2007**	Y	Y	Y	Y	Y		
**Nieder et al.**	Y			Y	Y		
**Sperduto et al.**	Y	Y	Y	Y	Y		Y
**Ahn et al.**	Y	Y	Y		Y	Y	Y
**Marko et al.**	Y	Y	Y		Y	Y	Y
**Le Scodan et al. 2012**	Y	Y	Y		Y		
**Niwińska et al.**		Y	Y		Y		
**Rades et al.**	Y		Y		Y		Y

Y, yes (presented in study);

### Validation

Our validation set included 642 Asian *de novo* metastatic breast cancer patients with a median age of 53 years (range, 24–94). Patient characteristics are reported in Table 3. Over a follow-up period of 1267.6 person-years, 492 patients had died and the median survival time was 19 months (95% CI, 16.5–21.5). The 1-year, 2-year and 3-year survival rates were 62%, 43% and 31% respectively. Half of the patients had more than one metastatic site involved and the majority did not receive any surgery or radiotherapy. Chemotherapy and hormone therapy were administered to 53% and 32% of the study population respectively. Among the 87 NUH patients with comorbidity data, hypertension (30%) and diabetes (23%) were the most common medical conditions. Less than 10% of this group was suffering from coronary heart disease (7%), stroke (2%), chronic obstructive pulmonary disease (3%) and renal failure (1%) and 6% of the patients have more than two comorbidities.

**Table 3:** Characteristics of *de novo* metastatic breast cancer patients identified at NUH, TTSH and UMMC, 2000–2010.

		UMMC	NUH	TTSH	Overall
Total		266 (41.4%)	156 (24.3%)	220 (34.3%)	642
Median Survival in months (95% CI)		14.0 (11.7–16.3)	28.0 (20.9–35.1)	18.0 (12.2–23.8)	19.0 (16.5–21.5)
Median age at diagnosis in years (range)		50 (24–83)	53 (28–80)	58 (30–94)	53 (24–94)
Median tumor size in mm (range)		100 (5–300)	40 (2–210)	60 (2–200)	60 (2–300)
**Ethnicity**	Chinese	148 (55.6%)	95 (60.9%)	152 (69.1%)	395 (61.5%)
	Malay	88 (33.1%)	38 (24.4%)	39 (17.7%)	165 (25.7%)
	Indian	30 (11.3%)	12 (7.7%)	15 (6.8%)	57 (8.9%)
	Others	0 (0.0%)	11 (7.1%)	14 (6.4%)	25 (3.9%)
**Grade**	1	2 (0.8%)	5 (3.2%)	3 (1.4%)	10 (1.6%)
	2	53 (19.9%)	64 (41.0%)	40 (18.2%)	157 (24.5%)
	3	63 (23.7%)	70 (44.9%)	41 (18.6%)	174 (27.1%)
	Unknown	148 (55.6%)	17 (10.9%)	136 (61.8%)	301 (46.9%)
**ER status**	Negative	102 (38.3%)	51 (32.7%)	81 (36.8%)	234 (36.4%)
	Positive	116 (43.6%)	103 (66.0%)	129 (58.6%)	348 (54.2%)
	Unknown	48 (18.0%)	2 (1.3%)	10 (4.5%)	60 (9.3%)
**PR status**	Negative	104 (39.1%)	62 (39.7%)	130 (59.1%)	296 (46.1%)
	Positive	63 (23.7%)	92 (59.0%)	80 (36.4%)	235 (36.6%)
	Unknown	99 (37.2%)	2 (1.3%)	10 (4.5%)	111 (17.3%)
**HER2 status**	Negative	64 (24.1%)	71 (45.5%)	75 (34.1%)	210 (32.7%)
	Positive	77 (28.9%)	24 (15.4%)	57 (25.9%)	158 (24.6%)
	Equivocal	20 (7.5%)	12 (7.7%)	17 (7.7%)	49 (7.6%)
	Unknown	105 (39.5%)	49 (31.4%)	71 (32.3%)	225 (35.0%)
**Site(s) of metastases**	Bone only	57 (21.4%)	25 (16.0%)	46 (20.9%)	128 (19.9%)
	Lung only	45 (16.9%)	11 (7.1%)	30 (13.6%)	86 (13.4%)
	Liver only	22 (8.3%)	9 (5.8%)	20 (9.1%)	51 (7.9%)
	Brain only	5 (1.9%)	2 (1.3%)	2 (0.9%)	9 (1.4%)
	Soft tissue only	5 (1.9%)	0 (0.0%)	3 (1.4%)	8 (1.2%)
	Other organ only	2 (0.8%)	1 (0.6%)	3 (1.4%)	6 (0.9%)
	Multiple sites	118 (44.4%)	104 (66.7%)	106 (48.2%)	328 (51.1%)
	Unknown	12 (4.5%)	4 (2.6%)	10 (4.5%)	26 (4.0%)
**Surgery**	No surgery	155 (58.3%)	84 (53.8%)	165 (75.0%)	404 (62.9%)
	Mastectomy	111 (41.7%)	63 (40.4%)	51 (23.2%)	225 (35.0%)
	Breast conserving surgery	0 (0.0%)	9 (5.8%)	4 (1.8%)	13 (2.0%)
**Chemotherapy**	No	101 (38.0%)	77 (49.4%)	53 (24.1%)	231 (36.0%)
	Yes	164 (61.7%)	79 (50.6%)	94 (42.7%)	337 (52.5%)
	Unknown	1 (0.4%)	0 (0.0%)	73 (33.2%)	74 (11.5%)
**Radiotherapy**	No	115 (43.2%)	106 (67.9%)	129 (58.6%)	350 (54.5%)
	Yes	96 (36.1%)	45 (28.8%)	19 (8.6%)	160 (24.9%)
	Unknown	55 (20.7%)	5 (3.2%)	72 (32.7%)	132 (20.6%)
**Hormone therapy**	No	63 (23.7%)	95 (60.9%)	120 (54.5%)	278 (43.3%)
	Yes	121 (45.5%)	58 (37.2%)	29 (13.2%)	208 (32.4%)
	Unknown	82 (30.8%)	3 (1.9%)	71 (32.3%)	156 (24.3%)

**Figure 2:**
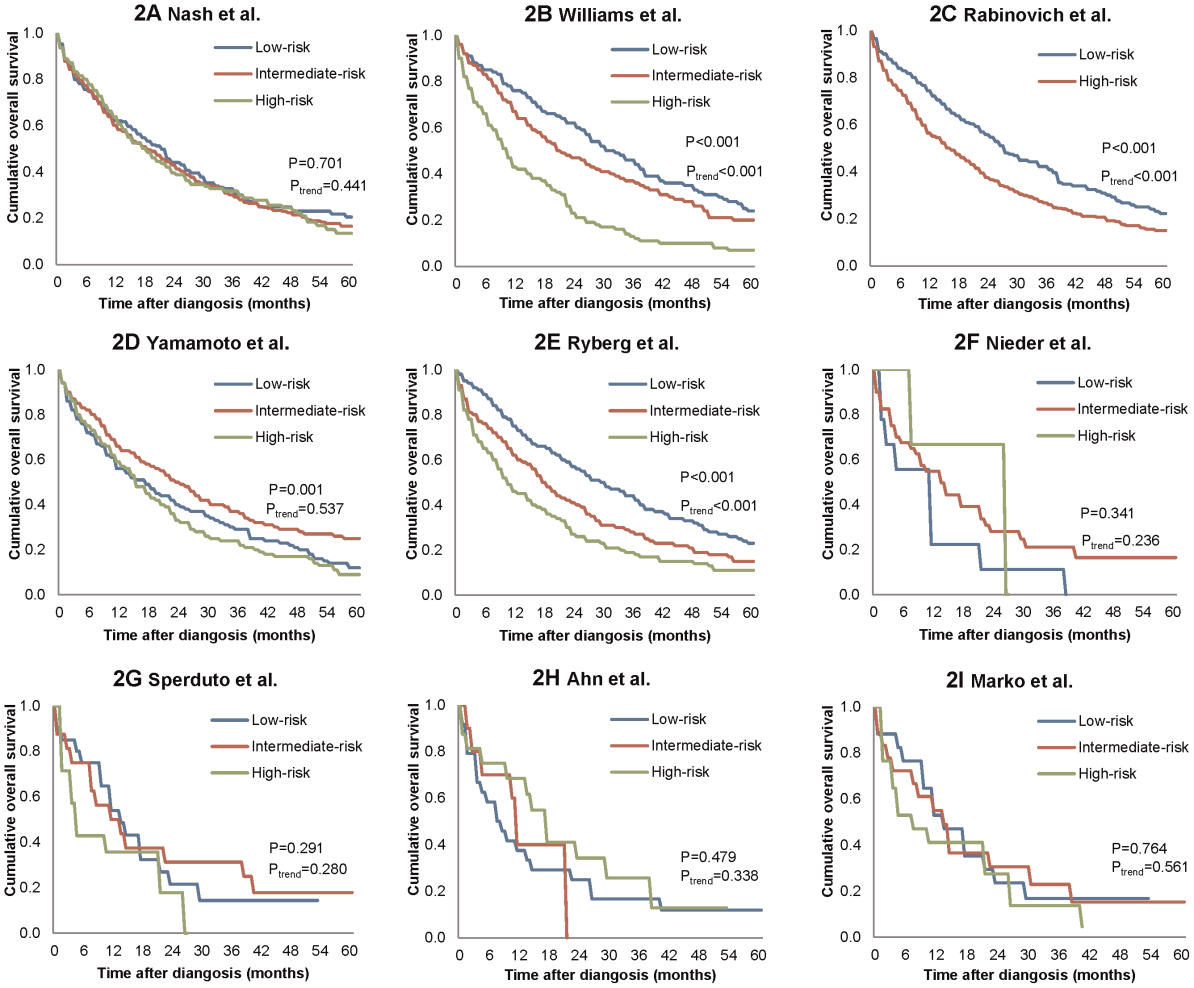
Kaplan Meier survival curves of low, intermediate and high-risk groups. Risk groups were defined by tertiles of risk scores of prediction models for patients with *de novo* metastatic breast cancer.

We validated all models that used Cox regression, with the exception of the models developed by Hortobagyi et al., Giordano et al., Le Scodan et al. and Rades et al. because the key predictors alkaline phosphatase (ALKP), circulating tumor cells (CTC), lymphocyte count and metastasis to spine were not available. Only Williams et al.'s, Yamamoto et al.'s, Rabinovich et al.'s and Ryberg et al.'s models were able to significantly discriminate between different risk groups in terms of overall survival based on log-rank test (Figure 2). The median survival for the low-risk group, intermediate-risk group and high-risk group classified according to Williams et al.'s model was 30 months, 21 months and 10 months respectively. For Rabinovich et al.'s model with two possible combinations, the median survival was 27 months and 16 months for the low and high risk groups. For Ryberg et al.'s model, the median survival was 29, 17 and 10 months respectively for the three groups. However the log-rank for trend test was not significant for Yamamoto et al.'s model as the median survival was 17 months for the low risk group, 24 months for the medium risk group and 15 months for the high risk group.

**Figure 3:**
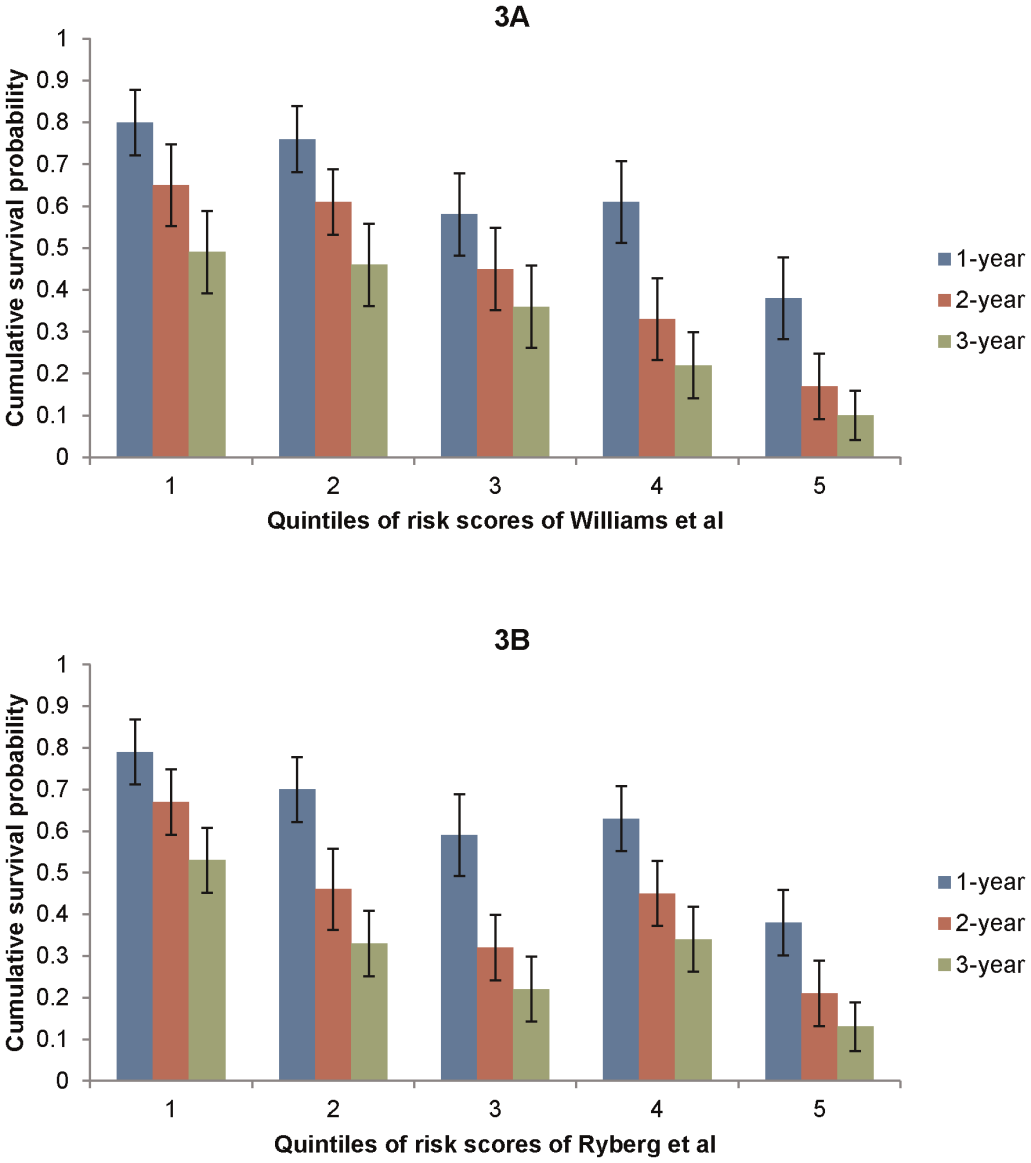
1-, 2- and 3-year cumulative survival probability for different risk groups. Risk groups were defined by quintiles of risk scores of Williams et al.'s and Ryberg et al.'s model. 1^st^ quintile is the group with the highest predicted survival probability and 5^th^ quintile is with the lowest predicted survival probability.

In our cohort, discrimination of the different models was poor to fair, with C-statistics ranging from 0.51 to 0.63 (Table 4). The model with the highest discriminatory ability was the model developed by Williams et al. (C-statistic 0.63, 95% CI 0.60–0.66), followed by Ryberg et al. (C-statistic 0.61, 95% CI 0.59–0.64). A notable decreasing trend of 1-year, 2-year and 3-year cumulative survival probabilities was observed for the five risk groups (quintiles, Figure 3). For Williams et al.'s model, the 3-year survival probabilities for the lowest and highest risk group were 49% (95% CI, 39%–58%) and 10% (95% CI, 4%–16%) respectively. For Ryberg et al.'s model, 3-year survival probabilities were 53% (95% CI, 45%–61) and 13% (95% CI, 7%–19%) for the low versus high risk groups respectively.

**Table 4:** Validation of selected models for prediction of survival of patients with *de novo* metastatic breast cancer.

Model	Number of subjects available for validation	Possible range of scores	Observed range of scores	C-statistic (95% CI)
Nash et al.	642	0.23–3.44	0.23–3.44	0.51 (0.48,0.53)
Williams et al.	571^a^	−2.00–32.00	1.23–32.00	0.63 (0.60,0.66)^d^
Rabinovich et al.	642	0.80–2.38	0.80–1.05	0.55 (0.53,0.57)
Yamamoto et al.	642	0.00–6.33	3.33–6.33	0.50 (0.48,0.53)
Ryberg et al.	642	0.00–50.00	0.00–25.00	0.61 (0.59,0.64)
Nieder et al.	52^c^	0.00–5.00	1.00–3.00	0.55 (0.48,0.61)
Sperduto et al.	50^b^^,^ c	0.00–4.00	1.50–4.00	0.56 (0.47,0.65)
Ahn et al.	50^b^^,^ c	0.00–325.00	0.00–138.00	0.56 (0.46,0.66)
Marko et al.	52^c^	0.00–375.00	44.50–108.60	0.55 (0.45,0.64)

a Patients with brain metastases excluded.

b Patients with equivocal Her2 status were excluded.

c Exclusively patients with brain metastasis.

d C-statistic for complete case analysis based on 297 patients was 0.63 (95% CI, 0.59–0.67).

## Discussion

Survival after *de novo* metastatic breast cancer, a relatively common condition among breast cancer patients in South East Asia, varies considerably. In this study, we showed that this highly variable prognosis can be predicted using currently available prediction rules, only to a certain extent in Asian patients. Overall, the prediction performance in the present series in Asia was not as good as in the original reports. Some of these prediction rules, which were identified through systematic review of the literature, used easily available clinical information such as age, hormone receptor status and site of metastasis. Some other models included biomarkers, which are not routinely available during the work up of breast cancer patients such as CTC and LDH.

We validated nine of the models in our Asian dataset and found that two models performed moderately well. In fact, with basic clinical information, (i.e. grade, ER status and site of metastasis), these models were able to classify patients as high risk and low risk. Based on risk scores calculated from Williams et al.'s and Ryberg et al.'s models, which included simple freely available clinical information, the difference of 3-year survival probability between the highest and lowest quintiles was close to 40%. Still, there was substantial overlap between the categories, and the current prediction rules were at best fairly able to discriminate between low and high risk patients (highest C-statistic = 0.63). Comparing to the other 3 models developed for all metastatic breast cancer patients, the models developed by Williams et al and Ryberg et al incorporated ER status and also grouped metastatic site into more categorizes. We were unable to validate the models which included advanced biomarkers, as this information was not routinely captured in our patients.

The inferior performance of the models in our Asian dataset as compared to the original report could be explained by unavailability of some predictors in our cohort and the fact that these indices/models were not specifically designed for *de novo* metastatic breast cancer. Another explanation could be that the Western derived models are not suitable for Asia setting. For example, in women with stage I–III breast cancer, Adjuvant!Online overpredicted survival by almost 7% and this overprediction was especially pronounced in younger women and women of Malay descent [50]. The underlying cause might be different distributions of age, tumor characteristics, competing risks and life styles factors. Several studies have reported that Asian breast cancer patients are more likely to be premenopausal, ER/PR-negative and HER2-positive [51], [52], [53]. Such differences could result in more skewed or more restricted range of prediction scores (Table 4).

Accuracy of predicting survival is crucial for women with *de novo* metastatic breast cancer as treatment varies widely, from no treatment at all, to removal of primary tumor and aggressive systemic treatment. The use of endocrine therapy and anti-HER2 drugs has been shown to prolong survival of metastatic patients.[54], [55], [56] Many randomized control trials have also reported significant survival benefit from modern chemotherapeutic agents, such as taxanes [57]. Recent studies have suggested that women who undergo surgery for *de novo* metastatic breast cancer have a significantly lower risk of death as compared to those who do not [16], [17], [18]. However the high proportion of patients not treated in our cohort or different response to treatment between Asian and Caucasian women may affect the usefulness of certain predictors such as hormone receptor status as well as the overall performance of the prediction models.

We acknowledge that our study suffers from limitations. The main limitation of the current study is the unavailability of certain clinical variables for prediction in our database such as performance status and LDH. Performance status, either recorded in Zubrod/ECOG/WHO or KPS, is a significant predictor in 11 indices/models. According to the development studies, 60% to 79% of their study population in fact had good performance status (Zubrod/ECOG/WHO = 0 or 1 or KPS≥70). Based on the results from a subset of patients with comorbidity data in our validation set, our assumption of patients to be generally fit may have resulted in some overestimation of predicted survival probabilities for a subset of patients. The number of CTC has been shown to be highly predictive for overall survival in patients with metastatic breast cancer [58], [59]. The CELLSEARCH test (Veridex, LLC, Raritan, NJ, USA) is the first and only clinically validated, FDA-cleared system for CTC assessment [60], [61]. However it is not routinely measured in Asia and is unlikely to be measured in future in low and middle income countries. The underperformance of models developed for brain metastasis maybe partially caused by the exclusion of non-treated patients in the development study, the lack of largest brain metastasis dimension and trastuzumab use in our validation dataset. Another limitation of our validation is the incomplete data of certain predictors. The pattern of missingness suggested missing at random and thus imputation is a better and more reasonable option than complete case analysis. The C-statistic for Williams et al's model from complete case analysis of 297 patients with grade, ER status and metastatic site(s) was 0.63 (95% CI, 0.59–0.67), which was very similar to the result from imputation (0.63, 95% CI, 0.60–0.66). However the standard errors and confidence intervals of the estimates might be too low as we ignored the uncertainty of imputed values by single imputation.

We conclude that existing prognostic models can only moderately predict survival of women with *de novo* metastatic breast cancer in the Asian setting. New models derived from a representative sample from an Asian population with different disease burden, would be able to accurately discriminate between patients with relatively good versus poor prognosis better.

## Supporting Information

Checklist S1 **PRISMA checklist.**
(DOC)

Figure S1 **PRISMA flowchart.**
(DOC)
