# Supplementary material for: Predicting Survival of De Novo Metastatic Breast Cancer in Asian Women: Systematic Review and Validation Study
Source: PLoS One. 2014 Apr 2;9(4):e93755. doi: 10.1371/journal.pone.0093755 (PMC3973579; doi:10.1371/journal.pone.0093755)
Supplement: Figure S1 — PRISMA flowchart. (DOC) [file pone.0093755.s002.doc]

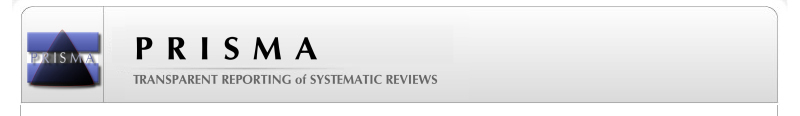
**PRISMA 2009 Flow Diagram**

**Screening**

**Included**

**Eligibility**

**Identification**

Records identified through database searching
(n = 1298)

Additional records identified through other sources
(n = 2)

Records after duplicates removed
(n = 1300)

Records screened
(n = 1300)

Records excluded
(n = 1250)

Full-text articles assessed for eligibility
(n = 50)

Full-text articles excluded, with reasons
(n = 34 )

- No prediction model/index developed (n=9)
- For any cancer, validated in breast (n=4)
- For any cancer, not validated in breast (n= 15)
- Validation studies of models for breast cancer (n=2)
- Other reasons (n=4)

Studies included in qualitative synthesis
(n = 16)

Studies included in quantitative synthesis

(Validation)
(n = 9)
